# The impact of heat on kidney stone presentations in South Carolina under two climate change scenarios

**DOI:** 10.1038/s41598-021-04251-2

**Published:** 2022-01-10

**Authors:** Jason Kaufman, Ana M. Vicedo-Cabrera, Vicky Tam, Lihai Song, Ethan Coffel, Gregory Tasian

**Affiliations:** 1 Perelman School of Medicine at the University of Pennsylvania, Philadelphia, PA USA; 2 Institute of Social and Preventive Medicine, University of Bern, Bern, Switzerland; 3 Oeschger Center for Climate Change Research, University of Bern, Bern, Switzerland; 4 Data Science and Biostatistics Unit, Department of Biomedical and Health Informatics, Children’s Hospital of Philadelphia, Philadelphia, PA USA; 5 Department of Geography and the Environment, Syracuse University, Syracuse, NY USA; 6 Division of Pediatric Urology, Children’s Hospital of Philadelphia, Philadelphia, PA USA; 7 Departments of Surgery and Biostatistics, Epidemiology, and Informatics, Perelman School of Medicine at the University of Pennsylvania, Philadelphia, PA USA

**Keywords:** Renal calculi, Climate-change impacts

## Abstract

The risk of kidney stone presentations increases after hot days, likely due to greater insensible water losses resulting in more concentrated urine and altered urinary flow. It is thus expected that higher temperatures from climate change will increase the global prevalence of kidney stones if no adaptation measures are put in place. This study aims to quantify the impact of heat on kidney stone presentations through 2089, using South Carolina as a model state. We used a time series analysis of historical kidney stone presentations (1997–2014) and distributed lag non-linear models to estimate the temperature dependence of kidney stone presentations, and then quantified the projected impact of climate change on future heat-related kidney stone presentations using daily projections of wet-bulb temperatures to 2089, assuming no adaptation or demographic changes. Two climate change models were considered—one assuming aggressive reduction in greenhouse gas emissions (RCP 4.5) and one representing uninibited greenhouse gas emissions (RCP 8.5). The estimated total statewide kidney stone presentations attributable to heat are projected to increase by 2.2% in RCP 4.5 and 3.9% in RCP 8.5 by 2085–89 (vs. 2010–2014), with an associated total excess cost of ~ $57 million and ~ $99 million, respectively.

## Introduction

Kidney stone disease (nephrolithiasis) is a painful condition affecting roughly one in eleven Americans, the incidence of which has increased in the last 20 years, particularly among women and adolescents^1,2^. Kidney stone disease is a disorder of mineral metabolism that is punctuated by acute symptomatic events that are usually caused by movement of a stone to the ureter and may be recurrent over the lifetime. It is well established that high ambient temperatures increase the risk of developing kidney stone disease and presenting with acute, symptomatic stones^3–5^. The evidence in support of the relationship between ambient climate and kidney stone disease first arose from observations that kidney stone presentations increase in warmer months, and that there is a North to South increase in kidney stone incidence in the United States^6^. More recently, our group and others have more precisely defined the relationship between hot days and kidney stone presentations, which is characterized by a short lag of up to 2 days and heterogeneity by geographic region and sex^5,7,8^. One proposed mechanism is that higher evaporative water losses leads to more concentrated urine, creating an environment in which crystallization of calcium, oxalate, uric acid, and phosphate is more likely^9,10^. This exact mechanism, however, has not been elucidated.

As global ambient temperatures increase from climate change, it is expected that the prevalence of kidney stone disease and associated acute kidney stone presentations will follow. The current study aims to quantify the attribute risk and associated cost of kidney stone presentations as a function of heat and humidity under two scenarios of climate change in South Carolina. By modeling two scenarios of future climate change—one more aggressive, one more conservative—we can also compare how the burden of kidney stone disease as reflected by acute presentations may be affected by climate policy.

## Methods

### Study design

We performed a two-step study to project the impact of heat on future kidney stone presentations in South Carolina. As a model state, South Carolina offers insight into the effect that climate change will have on a region with an already high disease burden. South Carolina lies within the “kidney stone belt” of the Southeast US, a region with a higher incidence of kidney stone disease thought to be secondary to both diet and higher temperatures^11^. First, we performed an aggregated case-crossover study to determine the relationship between historic daily state-wide mean wet-bulb temperatures (WBT) and kidney stone presentations in South Carolina from 1997 to 2014 (adapted from Vicedo-Cabrera et al.^8^). Next, we projected the heat-related number of kidney stone presentations and associated cost to 2089 based on projected daily WBT under two climate change scenarios.

### Exposure

The exposure used in this study is daily mean WBT, a moist heat metric that accounts for both ambient heat and humidity. We previously reported that wet-bulb temperature is the most accurate temperature metric for predicting kidney stone presentations in South Carolina^12^. The reason for this higher performance is thought to be due to the opposing effect of temperature and humidity on insensible water loss. Insensible water losses increase in higher temperatures, but decrease in higher relative humidities^6,12,13^. Observed daily mean WBT was obtained by averaging county-level measures of WBT across South Carolina from the NASA Land Data Assimilation Systems from January 1, 1997 to December 31, 2014^12,14^.

Projections of future WBT were generated by Dr. Ethan Coffel, Department of Geography and the Environment at Syracuse University^15^. Six predictive datasets were considered: three general circulation models (GCMs) (Access 1-0, BCC-esm, and CNRM-cm5) forced by two representative concentration pathways (RCPs) of global climate change (RCP 4.5 and RCP 8.5). The GCMs were selected to capture some variability within each RCP, and were selected in consultation with the climate scientist on the team.

The RCPs represent different pathways of future climate change, depending on human factors such as land use change and efforts to mitigate climate change. RCP 4.5 and 8.5 lead to radiative forcing outcomes of 4.5 and 8.5 W/m^2^ by 2100, respectively, with higher values representing a greater greenhouse gas effect. GCMs project different trajectories in approaching that endpoint based on climatic factors including the atmosphere, oceans, and daily variations in weather^16^. Three GCMs were selected at random to capture a range of assumptions regarding day to day variation within each RCP, in consultation with the team’s climate scientist. Each projection dataset was calibrated to minimize long-term differences between historic and projection datasets, using methodology adapted from Hempel et al.^17^.

RCP 4.5 represents an “intermediate” future, with shifts to lower-emissions sources of energy, the use of carbon capture technology, prices on CO_2_ emissions, and an expansion of forest lands ^16,18^. RCP 8.5, conversely, represents a future with high population growth, high energy demand, little advancement towards greater efficiency, and a greater dependence on domestic, more easily accessible energy sources like coal which have a higher greenhouse gas footprint^19^. Under RCP 8.5, forest lands decrease and there are far fewer assumed policies to mitigate greenhouse gas emissions^19^. While RCP 4.5 represents more aggressive greenhouse gas mitigation strategies, RCP 8.5 represents a future with mostly uninhibited greenhouse gas emissions^16^. Comparing these two possible futures in this study, we can compare the projected effect that aggressive global climate policies, actions, or inactions could have on kidney stone presentations.

As the projection dataset covered the entire globe with a 2° × 2° grid, we divided South Carolina into five geographic regions (Supplementary Appendix A), and averaged the daily WBT at the center of these regions to generate a single future daily mean WBT projection for the state per projection.

### Outcome

The outcome was the number of index kidney stone presentations in South Carolina emergency rooms. For the first stage in which the temperature dependence of kidney stone presentations was modeled, the counts of kidney stone presentations from January 1, 1997 to December 31, 2014 was obtained from the South Carolina Medical Encounter Data and Financial Reports all-payer claims database. This database includes all emergency department visits, surgical procedures, and hospital admissions across South Carolina, with individual IDs and accompanying demographic data. Audits are regularly performed on this dataset to ensure 99.9% accuracy of diagnostic codes and 99.5% completion^20^. Kidney stone cases were identified as emergency room visits associated with a primary International Classification of Diseases, 9th and 10th Revision codes for kidney and urinary tract stones (ICD-9 codes 592.0, 592.1, 592.9, and 274.11; ICD-10 N20). For individuals with multiple encounters with these codes, only the earliest encounter was considered so as to not double-count multiple encounters related to a single kidney stone event. Cost per presentation was simplified as the mean total charges incurred by all patients presenting through the emergency room with a primary presentation of kidney stone disease from January 1, 1997 to December 31, 2014, without adjusting for inflation. This average cost per patient between within these dates was $9525.95.

### Statistical analysis

The first step consisted of the estimation of the exposure–response curve between daily WBT and kidney stone presentations using historical data and the method and modelling specifications described in Vicedo-Cabrera et al. Briefly, conditional quasi-Poisson regression with distributed lag non-linear models (DLNMs) was used to determine the non-linear and delayed association accounting up to 10 days after the exposure. We fitted a natural spline with two internal knots equally spaced in the log scale in the exposure-lag dimension. For the exposure–response dimension, we used a natural spline function, instead of the original quadratic b-spline, with internal knots at the 50th and 90th percentile of the temperature distribution. The use of natural splines allowed for the log-linear extrapolation of risk beyond the observed range of temperatures in the historical dataset, a critical consideration when projecting into years with temperatures higher than those ever observed in the historical period^21^.

In the second step, the overall cumulative exposure–response association was used to estimate the number of WBT-related kidney stone presentations in future periods using each of the six daily projections of WBT. The method used is described in detail in Vicedo-Cabrera et al.^21^. The total number of kidney stone presentations attributed to heat in each 5-year period between 2025 and 2089 (above the mean from 2010 to 2014) was computed by summing the daily temperature-related number of cases on days with WBT > 6.7 °C (defined as the temperature of minimum risk estimated from the exposure–response curve) (see Vicedo-Cabrera et al.^21^). Estimates were reported as average ensemble estimates across the 3 GCMs for each RCP. This modeling approach assumes no changes in population demographics or vulnerability (i.e., adaptation). That is, estimates reflect the impact on today’s population in South Carolina if exposed to a warmer climate consistent with these two climate change scenarios. As measures of uncertainty, 95% empirical confidence intervals (eCI) were estimated using Monte Carlo simulations with 1000 samples of coefficients of the association over each of the GCM-specific series, assuming a normal distribution of the estimates.

## Results

**Figure 1:**
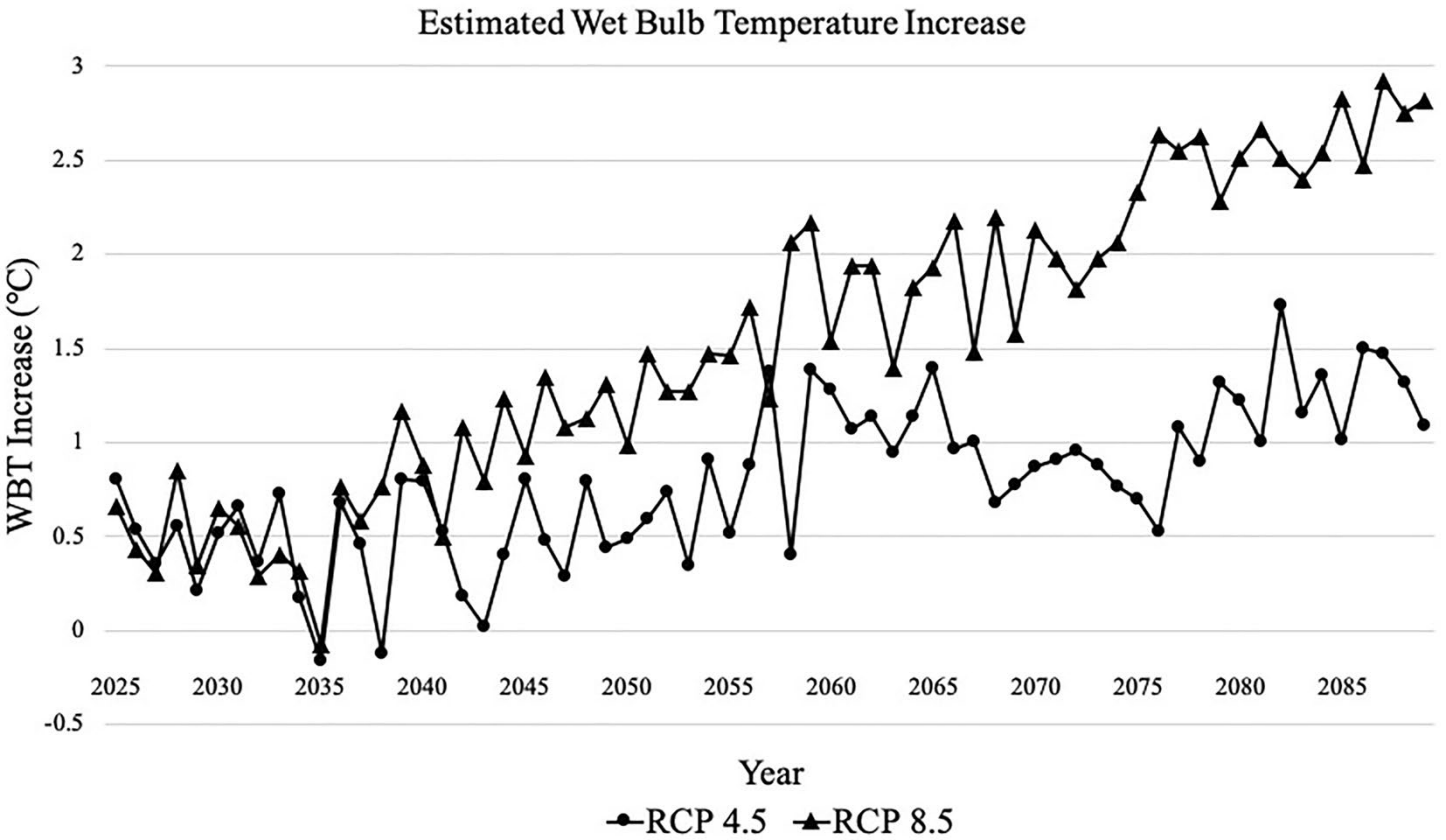
Increase in average annual WBT above average WBT between 1997 and 2014 for projected years in RCP 4.5 and RCP 8.5. Higher future WBT are associated with RCP 8.5 compared to RCP 4.5, due to greater projected future greenhouse gas concentration.

Both RCP 4.5 and 8.5 project an increase in temperatures from the 2010s to the 2080s, with RCP 8.5 projecting a larger increase in temperatures secondary to greater greenhouse gas emissions compared to RCP 4.5 (Fig. 1). Tables 1 and 2 describe characteristics of the historic and projected temperature datasets for both RCPs. RCP 4.5 projects a 2.3°C increase in mean temperature per 5-year period from 2010–2014 to 2085–2089, while RCP 8.5 projects a 3.6°C increase in the same time frame (Table 2). The number of days exceeding the historic maximum daily mean from 1997 to 2014 (26.0 °C) are also expected to increase over the course of the century, with far more days exceeding this historic maximum in RCP 8.5 (Table 2).

**Table 1:** Mean and maximum daily WBT for historic periods assessed in this study.

Historic year range	Mean daily WBT (°C) (IQR)	Maximum daily mean WBT (°C)
1997–1999	14.2 (8.4–20.6)	25.7
2000–2004	14.0 (8.0–21.0)	24.7
2005–2009	14.0 (8.1–20.6)	26.0
2010–2014	14.0 (7.6–20.9)	25.3

**Table 2:** Projected mean WBT and days exceeding historic maximum (26.0 °C) and the referent temperature (6.7 °C) in each projected 5-year period.

Projected year range	RCP 4.5			RCP 8.5		
	Mean daily WBT (°C) (IQR)	# Days above 26.0 °C	# Days above 6.7 °C	Mean daily WBT (°C) (IQR)	# Days above 26.0 °C	# Days above 6.7 °C
2025–2029	14.5 (8.7–21.2)	0	1525	14.5 (8.8–21.1)	0	1550
2030–2034	14.5 (8.7–21.1)	0	1526	14.4 (8.9–21.1)	0	1543
2035–2039	14.3 (8.3–21.3)	0	1490	14.6 (8.5–21.6)	0	1515
2040–2044	14.4 (8.3–21.3)	0	1496	14.9 (8.9–21.5)	0	1572
2045–2049	14.6 (8.5–21.6)	0	1527	15.2 (9.6–21.9)	0	1586
2050–2054	14.6 (8.6–21.6)	0	1536	15.3 (9.3–22.1)	0	1591
2055–2059	14.9 (9.1–22.0)	0	1565	15.7 (10.1–22.6)	4	1624
2060–2064	15.1 (9.0–21.9)	0	1565	15.7 (9.9–22.6)	5	1620
2065–2069	15.0 (9.1–21.7)	0	1564	15.9 (9.5–22.7)	8	1642
2070–2074	14.9 (8.8–22.0)	0	1576	16.0 (10.2–22.7)	7	1664
2075–2079	14.9 (8.6–22.2)	0	1516	16.5 (10.6–23.2)	23	1703
2080–2084	15.3 (9.2–22.4)	0	1594	16.5 (10.7–23.3)	46	1687
2085–2089	15.3 (9.3–22.2)	0	1576	16.8 (10.7–23.8)	69	1665

**Figure 2:**
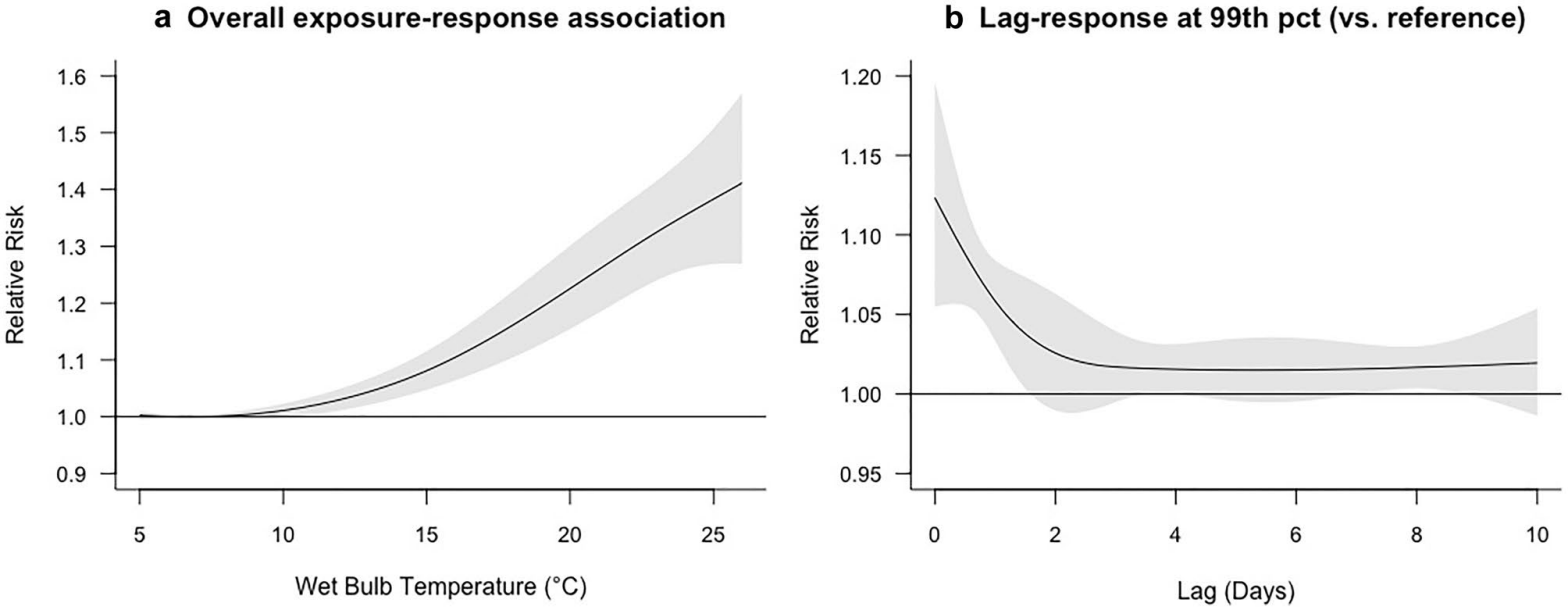
(**a**) The overall cumulative exposure–response relationship between daily WBT cumulated over 10-day lag period and kidney stone presentations, relative to 6.7 °C. (**b**) The lag-response relationship shows the distribution of risk over 10 days following a daily WBT at the 99th percentile, relative to the referent temperature of 6.7 °C.

The risk of kidney stone presentations increases non-linearly during the 10 days following a daily WBT greater than the referent temperature of 6.7 °C with no evidence of a ceiling effect at higher temperatures (Fig. 2a). Risk increases with higher temperatures and is greatest in the 2 days following a temperature event (Fig. 2b). The increase in heat-related kidney stone events (above the baseline from 2010 to 2014) from 2025 to 2089 is 5938 (95% CI 3730–9418) for RCP 4.5 and 10,431 (95% CI 6724–15,581) for RCP 8.5 (Table 3). The total cost attributable to these excess kidney stones from 2025–2089 is $56,565,091 for RCP 4.5 and $99,365,184 for RCP 8.5 (Table 3). The increase in the proportion of heat-related kidney stone presentations per 5 year period above the 2010–2014 baseline is 0.62% and 0.77% in the 2025–2029 period and 2.2% and 3.9% in the 2085–2089 period (RCP 4.5 and 8.5, respectively).

**Table 3:** Increase in the number of kidney stones attributed to heat (defined as WBT above 6.7 °C) and associated charges projected under RCP 4.5 and RCP 8.5 by 5-year interval between 2025 and 2089, versus 2010–2014 baseline.

Years	RCP 4.5		RCP 8.5	
	Increase in heat-related kidney stones presentations (95% CI)	Associated excess charges	Increase in heat-related kidney stones presentations (95% CI)	Associated excess charges
2025–2029	212 (61–396)	$2,019,501.40	258 (128–443)	$2,457,695.10
2030–2034	316 (179–450)	$3,010,200.20	348 (94–673)	$3,315,030.60
2035–2039	284 (154–457)	$2,705,369.80	250 (133–436)	$2,381,487.50
2040–2044	416 (270–565)	$3,962,795.20	548 (285–849)	$5,220,220.60
2045–2049	305 (94–514)	$2,905,414.75	541 (301–853)	$5,153,538.95
2050–2054	341 (181–522)	$3,248,348.95	695 (413–1078)	$6,620,535.25
2055–2059	433 (192–782)	$4,124,736.35	801 (401–1290)	$7,630,285.95
2060–2064	601 (368–874)	$5,725,095.95	1011 (553–1674)	$9,630,735.45
2065–2069	607 (236–1025)	$5,782,251.65	1010 (597–1521)	$9,621,209.50
2070–2074	523 (245–1008)	$4,982,071.85	1102 (546–1846)	$10,497,596.90
2075–2079	499 (229–800)	$4,753,449.05	1110 (558–1854)	$10,573,804.50
2080–2084	615 (217–1094)	$5,858,459.25	1348 (720–2200)	$12,840,980.60
2085–2089	786 (289–1448)	$7,487,396.70	1409 (717–2256)	$13,422,063.55

## Discussion

This analysis generated a projection of emergent kidney stone presentations and related costs in South Carolina under two different scenarios of climate change, one scenario with high future greenhouse gas emissions (RCP 8.5) and one path assuming more aggressive future climate mitigation policies (RCP 4.5). This analysis suggests a total increase of 5938 emergent kidney stone presentations attributed to heat with an associated total cost of ~ $57 million from 2025 to 2089 in RCP 4.5, compared to a total projected increase of 10,431 emergent kidney stone presentations attributed to heat and a total cost of ~ $99 million from 2025 to 2089 in RCP 8.5. While it is impossible to predict with certainty how future policies will slow or hasten greenhouse gas emission and anthropogenic climate change, and to know exactly what future daily temperatures will be, this analysis suggests that an increased burden of kidney stone disease on healthcare systems attributed to climate warming is very likely. This analysis additionally demonstrates how much of that increased burden could be curbed with moderately aggressive climate action.

A landmark 2008 study by Brikowski, et al. estimated that the number of Americans living in a “high risk” zone for kidney stones will increase from 40% in 2000 to 56% in 2050. Since 2008, we have a greater understanding of the temperature dependence of nephrolithiasis, which allows for more precise estimates of the impact of climate change on kidney stone presentations. While the Brikowski study applied hypothesized linear and non-linear relationships between temperature and kidney stone disease to the entire US, it has since been demonstrated that the relationship between temperature and kidney stone disease varies by geographic location, and that neither the hypothesized linear nor non-linear models accurately reflect the true exposure risk curve^5^. In addition, it has been demonstrated that moist–heat metrics such as WBT model the relationship between climate and kidney stone presentation better than dry bulb temperature alone, with or without corrections for relative humidity^5,8^, as temperature and humidity contribute differently to the extent of evaporative water loss^12^. Though more limited in geographic scope, the current study improves on this seminal 2008 study in several ways. In this study, we empirically defined the precise relationship between heat, humidity, and kidney stone presentation using historic data congruent with the study location. We also used WBT projections that account for future day-to-day variation in heat and humidity to define and project kidney stone disease risk.

There are several important assumptions to consider when interpreting the results of this study. First, we assumed a constant population at risk of kidney stone events from year to year; in reality, South Carolina’s population will change over time. While population increase is likely (and thus our estimates may be an underestimate), it is also likely that population will adapt better as hot and humid days become more common, e.g. by spending more time in climate-controlled environments and drinking more fluids. We chose not to consider adaptation as efforts accurately model this adaptation are often overly simplistic^21,22^. In addition, the future effect of adaptation would likely vary by population characteristics and has not been well quantified^23^. We also applied a single risk curve for the entire population although the risk of temperature related kidney stone presentations is sexually dimorphic^8^. It is likely, however, that our population-wide estimates are accurate assuming a consistent population-wide distribution between males and females. Finally, South Carolina has recently experienced an observed rapid increased incidence in kidney stone disease, with a ~ 1% increase in mean annual incidence of kidney stone disease from 1997 to 2012, and a particularly rapid rise among adolescents^2^. We did not project future increases in non-heat related presentations given the uncertainty of future shifts in the epidemiology of the disease.

Limitations to this study include the uncertain causal inference in the temperature-dependence of kidney stone presentations. Second, our estimates of total costs were based only on the medical costs incurred by a patient at their emergency department visit with a kidney stone. We did not consider inflation or indirect costs such as loss of work. Thus, the actual “cost” to society of the increased incidence of kidney stone disease are likely far greater. Third, we only considered one geographic area. Our group previously reported that the shape of the exposure response curve differs by location. These results, therefore, should not be extrapolated to other parts of the United States or other areas of the globe. As more data on kidney stone presentations and other heat-related health outcomes become available, these projections will be possible at a larger scale.

Despite these limitations, our analysis is a model to conceptualize how the burden of kidney stone disease is expected to progress with climate change, and also how mitigations to greenhouse gas emissions can offset some of this burden. With climate change a modern reality, the US can expect warmer days overall and more frequent extreme heat events^24^. A recent analysis of the proposed resolutions at the 2021 United Climate Change Conference indicated a likely increase in mean global temperature to below 2 °C by 2100 with adherence to these resolutions^25^, an outcome just below the RCP 4.5 pathway represented in this study. As our analysis shows, under even a relatively conservative projection of climate change we can expect a higher incidence and cost of symptomatic kidney stones, particularly in the near future before any compensatory adaptive efforts.

As the research community continues to elucidate the impacts that climate change will have on the environment and human well-being, it is important to explore the burden of climate change on human health via physiologic stressors. Improving our understanding of this relationship will help health systems better prepare and encourage the public and policymakers to prioritize sustainable behaviors and policies to mitigate climate change.

## Supplementary Information

Supplementary Information.

## References

[1] Scales CD (2016). Urinary stone disease: Advancing knowledge, patient care, and population health. Clin. J. Am. Soc. Nephrol..

[2] Tasian GE (2016). Annual incidence of nephrolithiasis among children and adults in South Carolina from 1997 to 2012. Clin. J. Am. Soc. Nephrol..

[3] Soucie JM, Thun MJ, Coates RJ, McClellan W, Austin H (1994). Demographic and geographic variability of kidney stones in the United States. Kidney Int..

[4] Brikowski TH, Lotan Y, Pearle MS (2008). Climate-related increase in the prevalence of urolithiasis in the United States. PNAS.

[5] Tasian GE (2014). Daily mean temperature and clinical kidney stone presentation in five US metropolitan areas: A time-series analysis. Environ. Health Persp..

[6] Fakheri RJ, Goldfarb DS (2011). Ambient temperature as a contributor to kidney stone formation: Implications of global warming. Kidney Int..

[7] Chi BH (2017). Daily mean temperature and urolithiasis presentation in six cities in Korea: Time-series analysis. J. Korean Med. Sci..

[8] Vicedo-Cabrera AM, Goldfarb DS, Kopp RE, Song L, Tasian GE (2020). Sex differences in the temperature dependence of kidney stone presentations: A population-based aggregated case-crossover study. Urolithiasis.

[9] Masterson JH (2013). Changes in urine parameters after desert exposure: Assessment of stone risk in United States Marines transiently exposed to a desert environment. J. Urol..

[10] Eisner BH (2012). The effects of ambient temperature, humidity and season of year on urine composition in patients with nephrolithiasis. BJU Int..

[11] Sas DJ, Hulsey TC, Shatat IF, Orak JK (2010). Increasing incidence of kidney stones in children evaluated in the emergency department. J. Pediatr..

[12] Ross ME (2018). Assessment of the combination of temperature and relative humidity on kidney stone presentations. Environ. Res..

[13] Budd GM (2008). Wet-bulb globe temperature (WBGT)—Its history and its limitations. J. Sci. Med. Sport.

[14] Mitchell KE (2004). The multi-institution North American Land Data Assimilation System (NLDAS): Utilizing multiple GCIP products and partners in a continental distributed hydrological modeling system. J. Geophys. Res. Atmos..

[15] Coffel ED, Horton RM, de Sherbinin A (2018). Temperature and humidity based projections of a rapid rise in global heat stress exposure during the 21st century. Environ. Res. Lett..

[16] Pachauri RK, Meyer LA (2014). IPCC, 2014: Climate Change 2014: Synthesis Report. Contribution of Working Groups I, II and III to the Fifth Assessment Report of the Intergovernmental Panel on Climate Change.

[17] Hempel S, Frieler K, Warszawski L, Schewe J, Piontek F (2013). A trend-preserving bias correction—The ISI-MIP approach. Earth Syst. Dyn..

[18] Thomson AM (2011). RCP4.5: A pathway for stabilization of radiative forcing by 2100. Clim. Change.

[19] Riahi K (2011). RCP 8.5—A scenario of comparatively high greenhouse gas emissions. Clim. Change.

[20] South Carolina Data Oversight Council (2014). Principles and Protocol for the Release of Health Care Data. South Carolina State Documents Depository.

[21] Vicedo-Cabrera AM, Sera F, Gasparrini A (2019). Hands-on tutorial on a modeling framework for projections of climate change impacts on health. Epidemiology.

[22] Gosling SN (2017). Adaptation to climate change: A comparative analysis of modeling methods for heat-related mortality. Environ. Health Persp..

[23] Morefield PE, Fann N, Grambsch A, Raich W, Weaver C (2014). Heat-related health impacts under scenarios of climate and population change. Int. J. Environ. Res..

[24] Luber G, McGeehin M (2008). Climate change and extreme heat events. Am. J. Prev. Med..

[25] Ou Y (2021). Can updated climate pledges limit warming well below 2 °C?. Science.

