# Supplementary material for: The impact of heat on kidney stone presentations in South Carolina under two climate change scenarios
Source: Sci Rep. 2022 Jan 10;12:369. doi: 10.1038/s41598-021-04251-2 (PMC8748744; doi:10.1038/s41598-021-04251-2)

Appendix A. Geographic midpoints (black dots) averaged to generate a single projected daily WBT for the entire state of South Carolina.


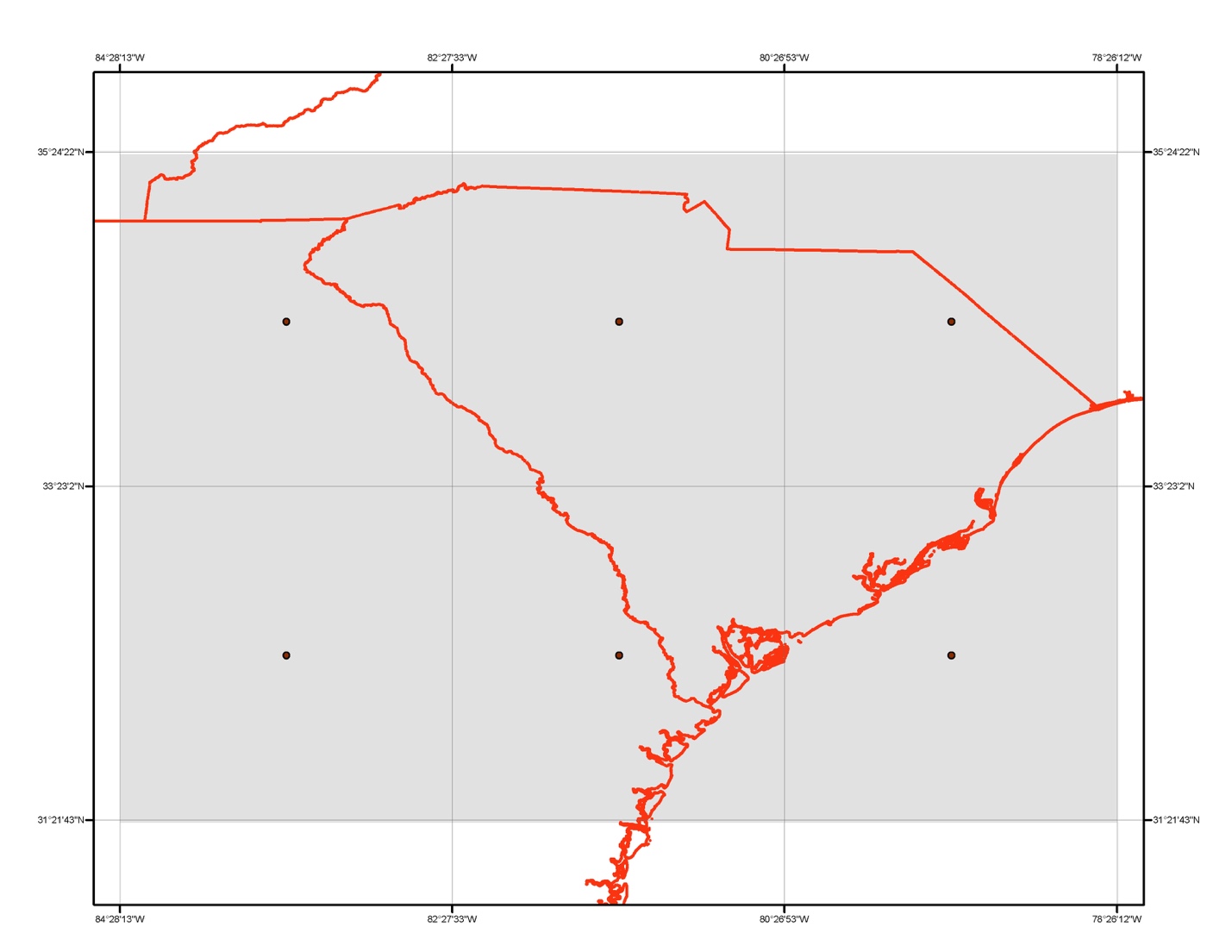

Supplement: Supplementary file 1 — Supplementary Information. [file 41598_2021_4251_MOESM1_ESM.docx]
